# Why are there more women than men on peritoneal dialysis in Brazil?

**DOI:** 10.1590/2175-8239-JBN-2025-0128en

**Published:** 2025-08-15

**Authors:** Fabiana Baggio Nerbass, Viviane Calice-Silva

**Affiliations:** 1 Fundação Pró-Rim, Joinville, SC, Brazil.; 1 Universidade da Região de Joinville, Joinville, SC, Brazil.

Dear Editor,

The curiosity surrounding the following question – “Why are there more women than men on peritoneal dialysis in Brazil?” – first emerged during the analysis of data from the Brazilian Dialysis Registry, which included nearly 25,000 incident dialysis patients between 2011 and 2022. As widely observed on a global level, we found a higher proportion of men undergoing dialysis (59%). However, one specific finding did not draw our attention at first: the higher proportion of women initiating renal replacement therapy with peritoneal dialysis (PD) compared to men (4.4% vs. 3.5%; p = 0.03)^1^. This finding merely reaffirmed what had already been observed in the large BRAZPD cohort in the first decade of the 2000s, in which, among more than 7,000 incident patients, 52% were female^2^ – a scenario also observed in our center.

However, our curiosity was heightened when we realized that this difference was not replicated worldwide. Major international reports, such as those from the US and Europe, do not support this disparity^3,4^. In both registries, an equally higher proportion of men is observed across hemodialysis and peritoneal dialysis modalities.

Given this context, a lack of recent epidemiological data on the national situation prevented us from assessing whether this trend was continuing. A recent study addressed this gap by analyzing the use of PD within the Brazilian public healthcare system – the main source of funding for this modality – over a 10-year period. From 2014 to 2023, women constituted the majority, with a prevalence ranging from 52% to 54%^5^.

The persistence of this difference between the sexes in PD in Brazil, in contrast to international data, may reflect a multifactorial interaction involving demographic, sociocultural, and institutional aspects. Below, we discuss some factors that may contribute to this phenomenon.

It is well recognized that women seek healthcare services more frequently than men, which, alongside a higher proportion of women in the early stages of CKD, may favor the planned initiation of dialysis and, consequently, the indication of PD as the initial modality.

In addition, the traditional caregiving role often assigned to women in our culture may, paradoxically, result in greater family support when they find themselves at vulnerability. This support may serve as an important facilitator for adherence to home-based treatment, which requires engagement and support from the care network.

Finally, it is possible to consider the existence of a clinical or institutional bias in the provision of this modality, based on the perception not always evidence-based – that women are better suited to managing home-based treatment. This perception may be associated with the idea that women demonstrate greater commitment to self-care and therapeutic routines, which could consciously or unconsciously influence the indication of PD.

These hypotheses, which transcend biological factors and involve social and structural dimensions, reinforce the need for further investigation. Understanding these nuances is essential to inform public policy planning that promotes equitable access to dialysis modalities, ensuring that therapeutic decisions reflect individual needs and preferences rather than stereotypes or invisible barriers.
