# Supplementary material for: Why are there more women than men on peritoneal dialysis in Brazil?
Source: J Bras Nefrol. 2025 Aug 15;47(4):e20250128. doi: 10.1590/2175-8239-JBN-2025-0128en (PMC12360828; doi:10.1590/2175-8239-JBN-2025-0128en)
Supplement: Translation

# Translation: Por que há mais mulheres do que homens em diálise peritoneal no Brasil?

**DOI:** 10.1590/2175-8239-JBN-2025-0128pt

**Authors:** Fabiana Baggio Nerbass, Viviane Calice-Silva

**Affiliations:** 1 Fundação Pró-Rim, Joinville, SC, Brasil.; 1 Universidade da Região de Joinville, Joinville, SC, Brasil.

Prezado Editor,

A curiosidade em torno da seguinte questão – “Por que há mais mulheres do que homens em diálise peritoneal no Brasil?” – surgiu inicialmente durante a análise dos dados do Registro Brasileiro de Diálise, que incluiu quase 25 mil pacientes incidentes em diálise entre 2011 e 2022. Como amplamente observado em nível global, verificamos uma maior proporção de homens em diálise (59%). No entanto, um dado específico não nos chamou a atenção de imediato: a maior proporção de mulheres iniciando a terapia renal substitutiva pela modalidade de diálise peritoneal (DP), em comparação aos homens (4,4% vs. 3,5%; p = 0,03)^1^. Esse achado apenas reafirmava o que havia sido observado anteriormente na grande coorte do BRAZPD, na primeira década dos anos 2000, na qual, entre mais de 7.000 pacientes incidentes, 52% eram do sexo feminino^2^ – cenário também observado em nosso serviço.

Nossa curiosidade, no entanto, se aguçou ao percebermos que essa diferença não se repetia globalmente. Grandes registros internacionais, como o americano e o europeu, não corroboram essa disparidade^3,4^. Em ambos, observa-se uma proporção igualmente maior de homens tanto na hemodiálise quanto na diálise peritoneal.

Diante disso, faltavam dados epidemi­ológicos mais recentes sobre a realidade nacional que nos permitissem avaliar se essa tendência persistia. Essa lacuna foi preenchida por um estudo recente, que analisou a utilização da DP no sistema público de saúde brasileiro — principal financiador da modalidade — ao longo de um período de 10 anos. Entre 2014 e 2023, as mulheres continuaram sendo maioria, com uma prevalência entre 52% e 54%^5^.

A persistência dessa diferença entre os sexos na DP no Brasil, em contraste com os dados internacionais, pode refletir uma interação multifatorial entre aspectos demográficos, socioculturais e institucionais. A seguir, discutimos alguns fatores que podem contribuir para esse fenômeno.

É reconhecido que as mulheres buscam os serviços de saúde com maior frequência do que os homens, o que, aliado a uma maior proporção de mulheres nos estágios iniciais da DRC, pode favorecer o início planejado da diálise e, por consequência, a indicação da DP como modalidade inicial.

Adicionalmente, o papel tradicional de cuidadora frequentemente atribuído à mulher em nossa cultura pode, paradoxalmente, resultar em maior suporte familiar quando ela se encontra em situação de vulnerabilidade. Esse suporte pode ser um facilitador importante para a adesão ao tratamento domiciliar, que exige engajamento e apoio da rede de cuidados.

Por fim, é possível considerar a existência de um viés clínico ou institucional na oferta da modalidade, baseado na percepção — nem sempre fundamentada — de que as mulheres seriam mais aptas a conduzir o tratamento domiciliar. Essa percepção pode estar associada à ideia de que as mulheres demonstram maior engajamento com o autocuidado e com as rotinas terapêuticas, o que poderia influenciar, consciente ou inconscientemente, a indicação da DP.

Essas hipóteses, que transcendem fatores biológicos e envolvem dimensões sociais e estruturais, reforçam a necessidade de investigações mais aprofundadas. Compreender essas nuances é essencial para subsidiar o planejamento de políticas públicas que promovam o acesso equitativo às modalidades dialíticas, assegurando que as decisões terapêuticas reflitam as necessidades e preferências individuais e não estereótipos ou barreiras invisíveis.
